# HIV-1 pseudoviruses constructed in China regulatory laboratory

**DOI:** 10.1080/22221751.2019.1702479

**Published:** 2019-12-20

**Authors:** Jianhui Nie, Weijin Huang, Qiang Liu, Youchun Wang

**Affiliations:** Division of HIV/AIDS and Sexually Transmitted Virus Vaccines, National Institutes for Food and Drug Control (NIFDC), Beijing, People’s Republic of China

**Keywords:** HIV-1, pseudovirus, neutralization assay, drug-resistance, mutation

## Abstract

To better evaluate HIV-1 vaccines and therapeutics, the National Institutes for Food and Drug Control of China developed a panel of HIV-1 pseudoviruses including 462 viral strains derived from China, covering the majority of contemporaneous subtypes and circulating recombinant forms. Compared with the standard pseudovirus panels derived from other countries/regions, the Chinese isolates are more susceptible to neutralization by the sera obtained in China, revealing the strain/subtype specificity. Some of these pseudoviruses have already been used for the evaluation of HIV vaccines and drug candidates in Chinese clinical trials. The pseudoviruses panel is widely shared with interested scientists involved in the research and development of vaccines and antiviral drugs against HIV-1 strains prevalent in China.

The National Institutes for Food and Drug Control (NIFDC) are affiliated with the National Medical Products Administration (NMPA) and are responsible for the development of new methods and references for the quality evaluation of food, medicines, and medical devices. To better evaluate the efficacy of HIV vaccines and therapeutics, the NIFDC developed a panel of HIV-1 pseudoviruses encompassing *env* variants representative of the Chinese region and studied their main characteristics. As summarized in this review, the region-specific pseudovirus panel has proven useful for the evaluation of HIV-1 vaccines and drug candidates.

## Construction of a panel of 124 strains of pseudovirus with the *env* gene from HIV-infected blood donors, drug users, and men who have sex with men (MSM)

**Figure 1.**
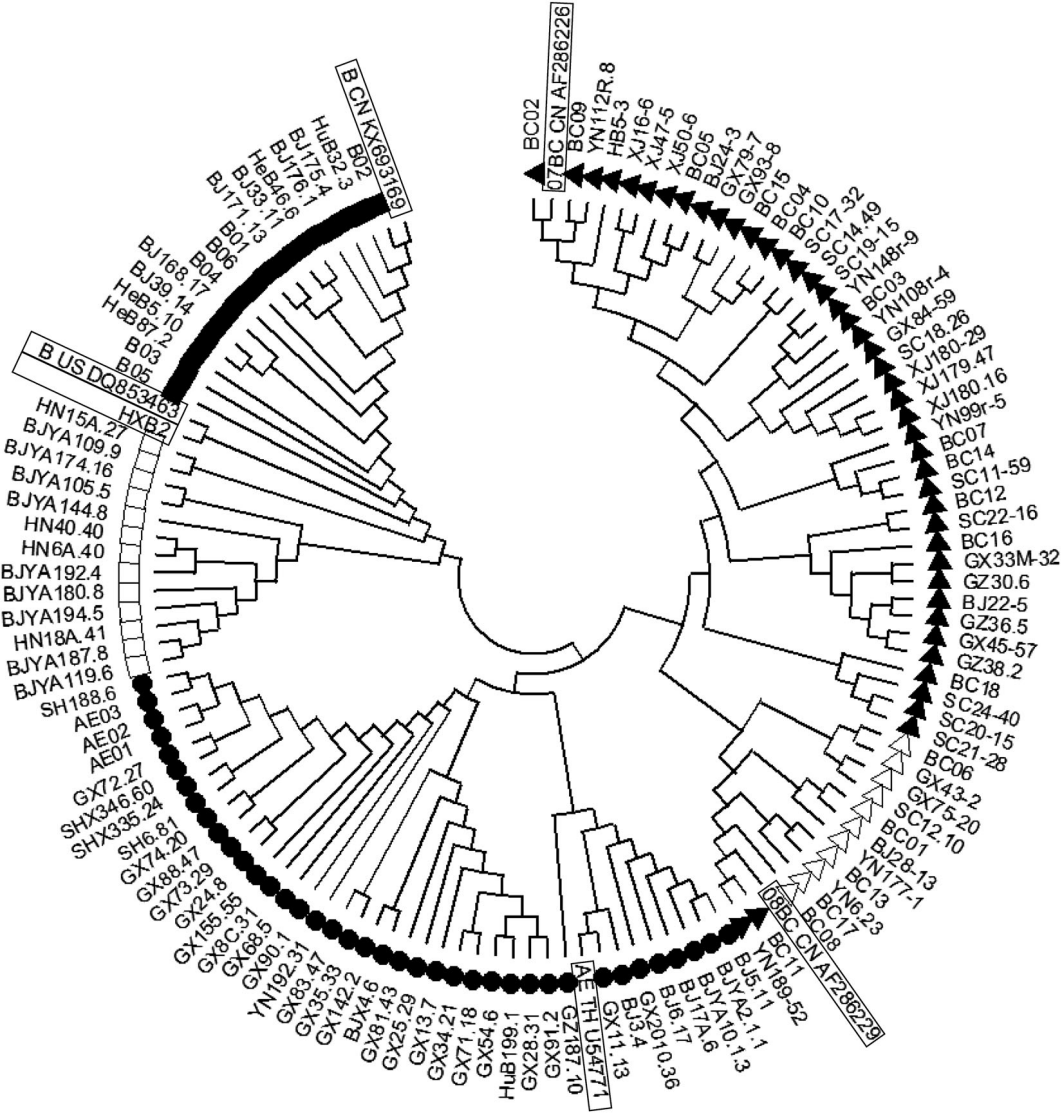
Phylogenetic tree of gp160 sequences derived from HIV-1 infections in China. Solid triangles (▴) represent subtype 07BC strains, hollow triangles (△) represent subtype 08BC strains, solid circles (●) represent 01AE strains, hollow squares (□) represent B strains solid squares (▪) represent Bʹ strains. The reference strain sequences are indicated by boxes.

The pseudovirus panels were constructed based on the neutralization assay for HIV-1 in Tzm-bl cells [1]. Briefly, RNA was extracted from HIV-1-positive plasma samples using a QIAmp Viral RNA kit (Qiagen, Hilden, Germany) and complementary DNAs (cDNAs) were generated using a Superscript First-Strand Synthesis kit (Invitrogen, Carlsbad, CA, USA). Six subtype-specific primer pairs were employed to amplify the *gp160* genes, which were directly inserted into pcDNA 3.1D/V5-His-TOPO (Invitrogen). Pseudoviruses were prepared by transfecting 293T cells with *env* expression plasmid and Env-deficient HIV-1 backbone vector (pSG3Δ*env*) using Lipofectamine 2000 reagent (Invitrogen). The 50% tissue culture infectious dose (TCID_50_) of each pseudovirus batch was determined in TZM-bl cells [2,3]. Finally, a total of 124 strains of pseudovirus were constructed including 42 strains from blood donors, 41 strains from drug users, and 41 strains from participants with confirmed sexually transmitted infections (17 from MSM) encompassing subtypes CRF01_AE (39), CRF07_BC (43), CRF08_BC (13), B (13), and B′ (16). The viral strains were isolated from the main HIV epidemic areas including Yunnan (20), Guangxi (30), Guangdong (1), Xinjiang (9), Henan (4), Sichuan (11), Shanghai (5), Hubei (3), Gansu (1), Hebei (7), and Beijing (32) (Figure 1) (Supplementary Table 1) [2–8].

**Figure 2.**
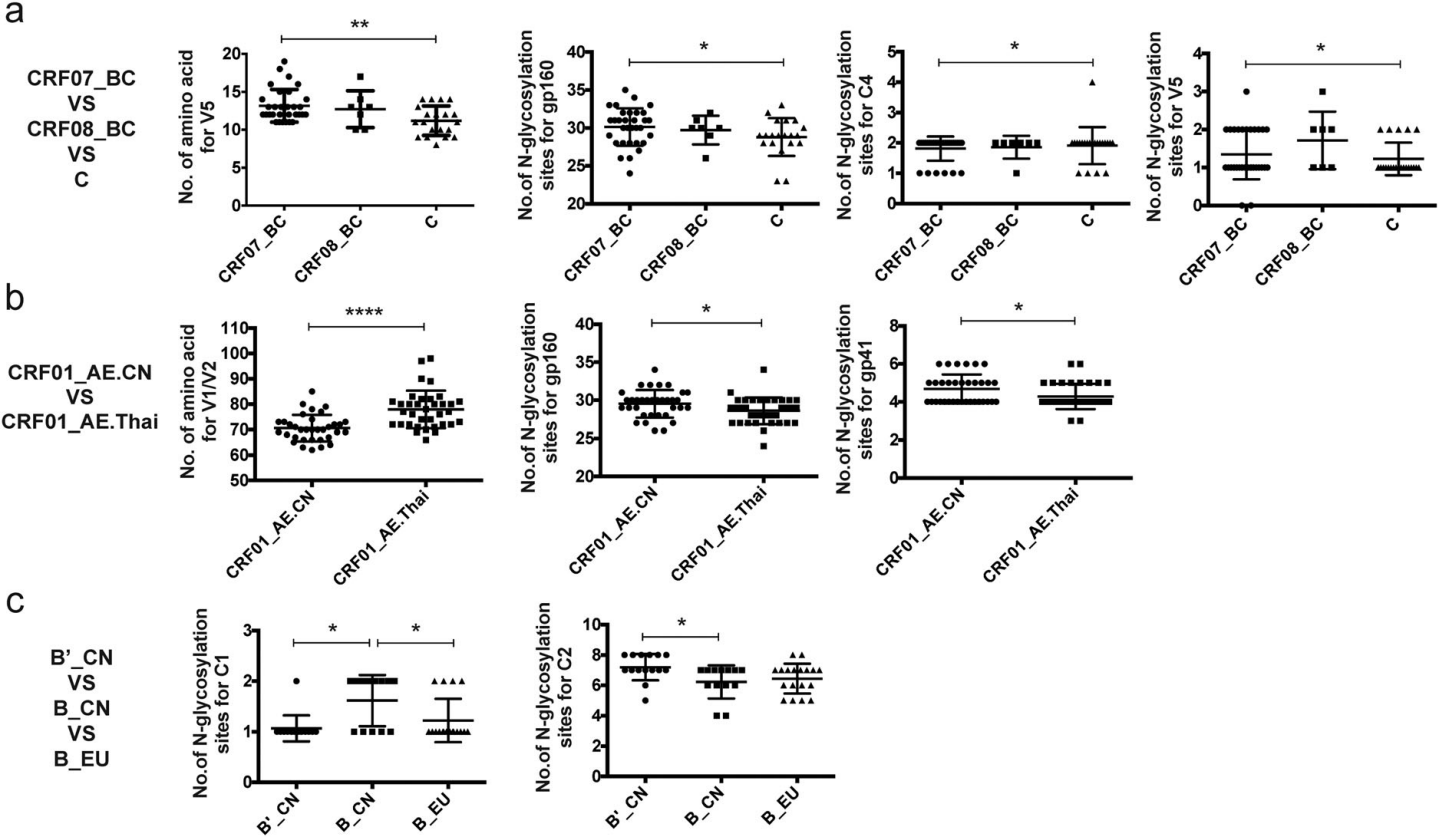
Comparison of the sequence characteristics of pseudoviruses between China and other countries. 1a. CRF_07BC (43 strains) and CRF_08BC (13 strains) from China and C subtype (20 strains) from other countries. 1b. CRF_01AE (39 strains) from China and CRF_01AE (35 strains) from Thailand. 1c. Bʹ (16 strains), B (13 strains) from China and B subtype (18 strains) from other countries. Comparative statistical methods: three groups were compared using one-way ANOVA combined with the Kruskal–Wallls test and two groups were compared using the *t* test. *p < 0.05, **p < 0.01, ****p < 0.0001.

Some distinct characteristics were found in the *env* genes by comparing the AE, BC, and B subtype pseudoviruses from China with those from other countries. Specifically, the V5 loop of CRF07_BC isolated in China was significantly longer than that of C subtype virus (*p* < 0.01), while the degree of Env glycosylation in CRF07_BC was significantly higher in Chinese isolates than in clade C Env proteins of viruses from other countries (*p* < 0.01), with differences in the numbers of glycosylation sites mostly found in V4 and V5 [5]. Moreover, the V1/V2 loops of CRF01_AE *env* strains in China were significantly shorter than those in strains of the same subtype from Thailand (*p* < 0.0001), with more extensive glycosylation observed in gp41 [3,9]. It has been reported that gp120 of subtypes C and A might expand over the course of infection and evolution [10] and it may be that the CRF01_AE strains in Thailand have evolved at a more rapid rate than in China. In addition, more extensive glycosylation was observed in C4 of the Env protein of B subtype virus in China compared with the Env proteins derived from other countries (*p* < 0.01) (Figure 2) [7].

**Figure 3.**
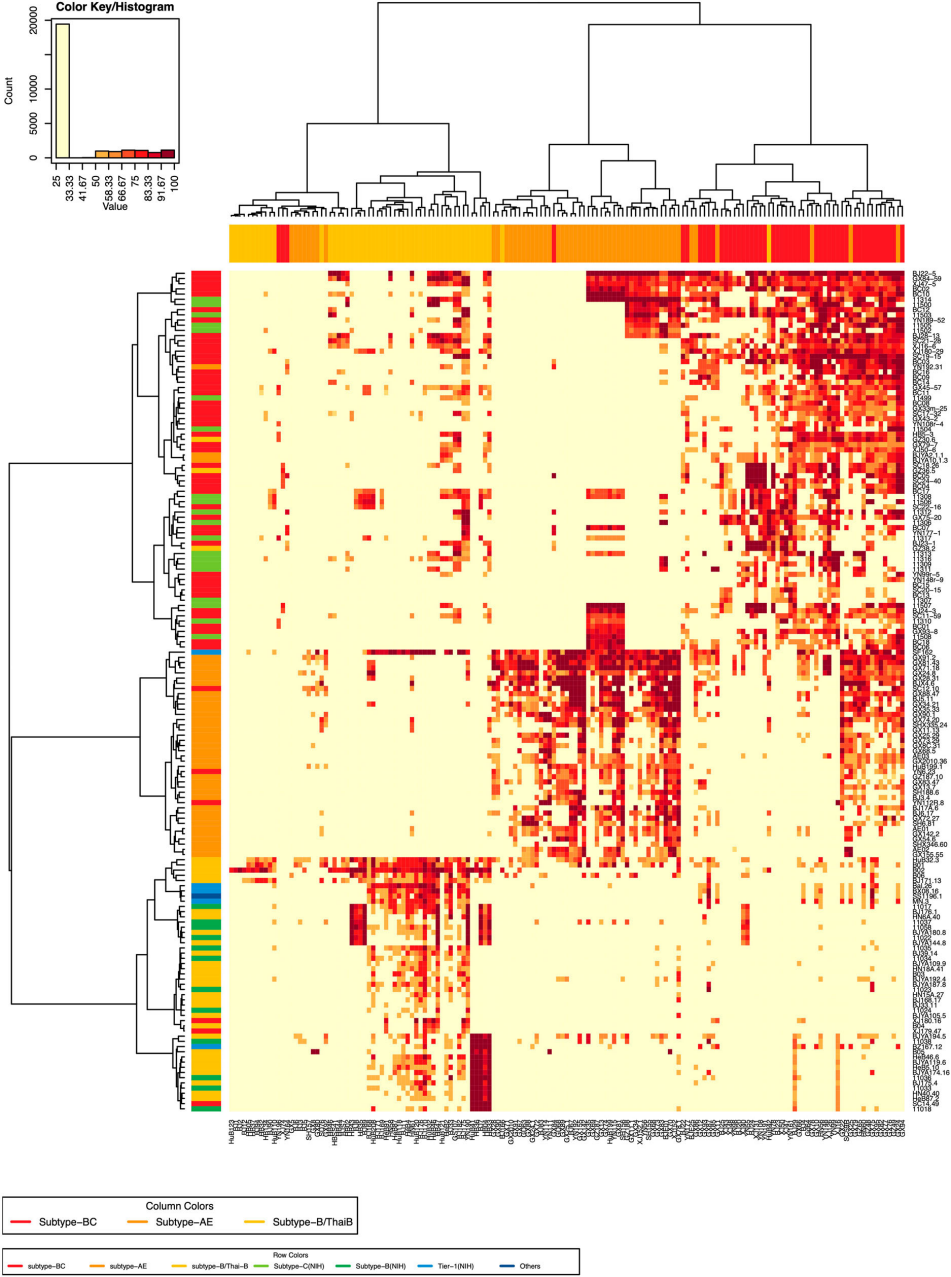
Heatmap to analyze neutralization sensitivity between the plasma pools and the pseudovirus panel. The heatmap programme was used to analyze the clustering patterns for pseudoviruses and plasma pools (https://www.hiv.lanl.gov/content/sequence/HEATMAP/heatmap_mainpage.html). This strategy clusters pseudoviruses based on their susceptibility to panels of plasmas, whilst simultaneously clustering plasmas based on their ability to neutralize a panel of pseudoviruses. The magnitude of neutralization (inhibition ratio of the relative light units) is denoted by colour. A colour palette was used to map the neutralization values to the colours: lower values are represented by less-saturated light colours, and higher neutralization values are represented by more-saturated dark colours. The subtype of the plasma samples was designated as the column colours in the upper margin. The subtype of the pseudoviruses was indicated as the row colours in the left margin.

We next investigated the neutralization sensitivity of 152 pseudoviruses (124 from China, 28 from other countries) with a panel of 157 HIV-1-positive plasma samples obtained from Chinese patients. The subtypes of the plasma samples were determined by partial *env* sequencing [11]. The plasma samples were derived from the most prevalent subtypes in China, including BC (53), B/Bʹ (52), and AE (52). We reported that the Chinese isolates were more susceptible to neutralization by the sera obtained in China [5,7]. When the 152 pseudoviruses were tested against the 157 plasma samples, relative subtype specificity could be observed (Figure 3). Based on the sensitivity of pseudoviruses to neutralizing sera [2,3,5,7], 124 pseudoviruses were classified into three tiers (Table 1), similar to Mascola’s recommendations [12]. When a pseudovirus could be neutralized by no less than 70% of HIV-1-positive plasma samples from the same subtype, it was grouped in Tier 1. Tier 2 pseudoviruses could be efficiently neutralized by 40%–70% of HIV-1 positive samples of the same type. Less than 40% neutralization-sensitive strains were classified into Tier 3. We established the Three-Tier strategy for pseudovirus evaluation based on the above pseudovirus stratification.

**Table 1.** Neutralization tiers of the pseudoviruses.

	Number of strains			
	CRF07/08_BC	CRF01_AE	B/B′	Total
Tier 1 (≥70%)^a^	10	4	4	18
Tier 2 (40%∼70%)	23	16	11	50
Tier 3 (<40%)	23	19	14	56
Total	56	39	29	124

Note: ^a^Tier 1 can be neutralized by 70% or more (≥70%) of the HIV-1-positive serum samples of the same subtype collected in China. Tier 2 pseudoviruses could be efficiently neutralized by 40%–70% HIV-1-positive samples of the same type. Less than 40% neutralization-sensitive strains were classified into Tier 3 pseudoviruses.

**Figure 4.**
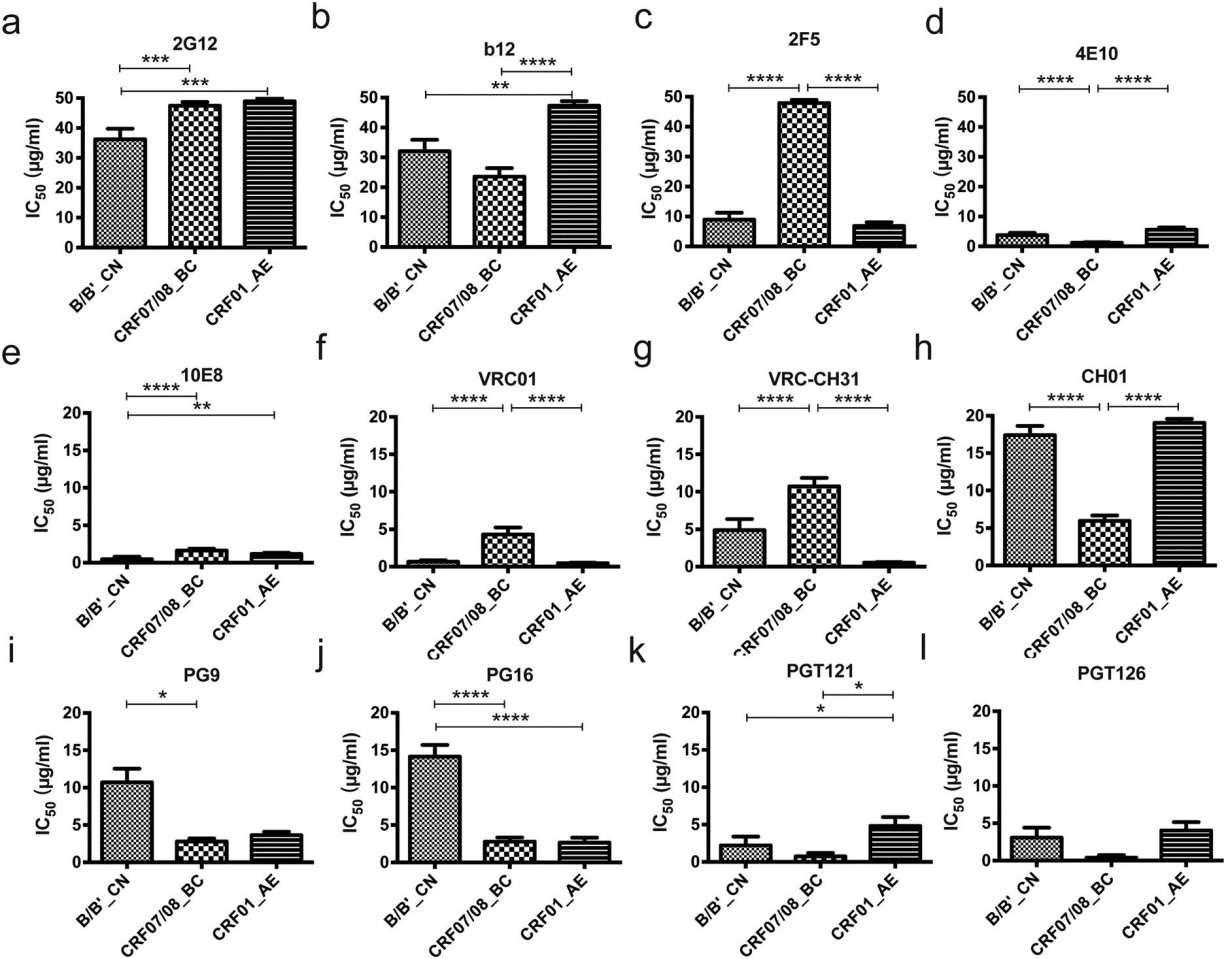
Analysis of the sensitivity of Chinese virus strains to known neutralizing mAbs. Twelve neutralizing mAbs targeting various epitopes: high-mannose patch-binding Abs (2G12 [2a]), CD4-binding site-binding Abs (b12 [2b], VRC01 [2f]), MPER-binding Abs (2F5 [2c], 4E10 [2d], 10E8 [2e]), Trimer apex-binding Abs (VRC-CH31 [2g], CH01 [2h], PG9 [2i], PG16 [2j]), V3-glycan-binding Abs (PGT121 [2k] and PGT126 [2l]) were used to neutralize CRF_07/08BC (55 strains), CRF_01AE (33 strains), and B/B′ (22 strains) from China. The geometric means are shown (three repeated runs). One-way ANOVA combined with the Kruskal–Wallls test were used to analyze the differences between types. *p < 0.05, **p < 0.01, ***p < 0.001, ****p < 0.0001.

Twelve well-characterized neutralizing monoclonal antibodies (mAbs) were also used to analyze the neutralizing susceptibility of 100 HIV-1 strains among 124 pseudoviruses. Six AE, seven B/Bʹ, and eleven BC strains were not included in the mAb assay, due to their identical pattern of neutralization susceptibility to some other strains in the plasma heatmap (Figure 3). The degree of neutralization varied depending on the subtype of the virus [2,3,5,7] (Figure 4).

## Analysis of the evolution of HIV-1 envelope protein under neutralizing antibody pressure in HIV-infected individuals (134 pseudovirus strains)

**Figure 5.**
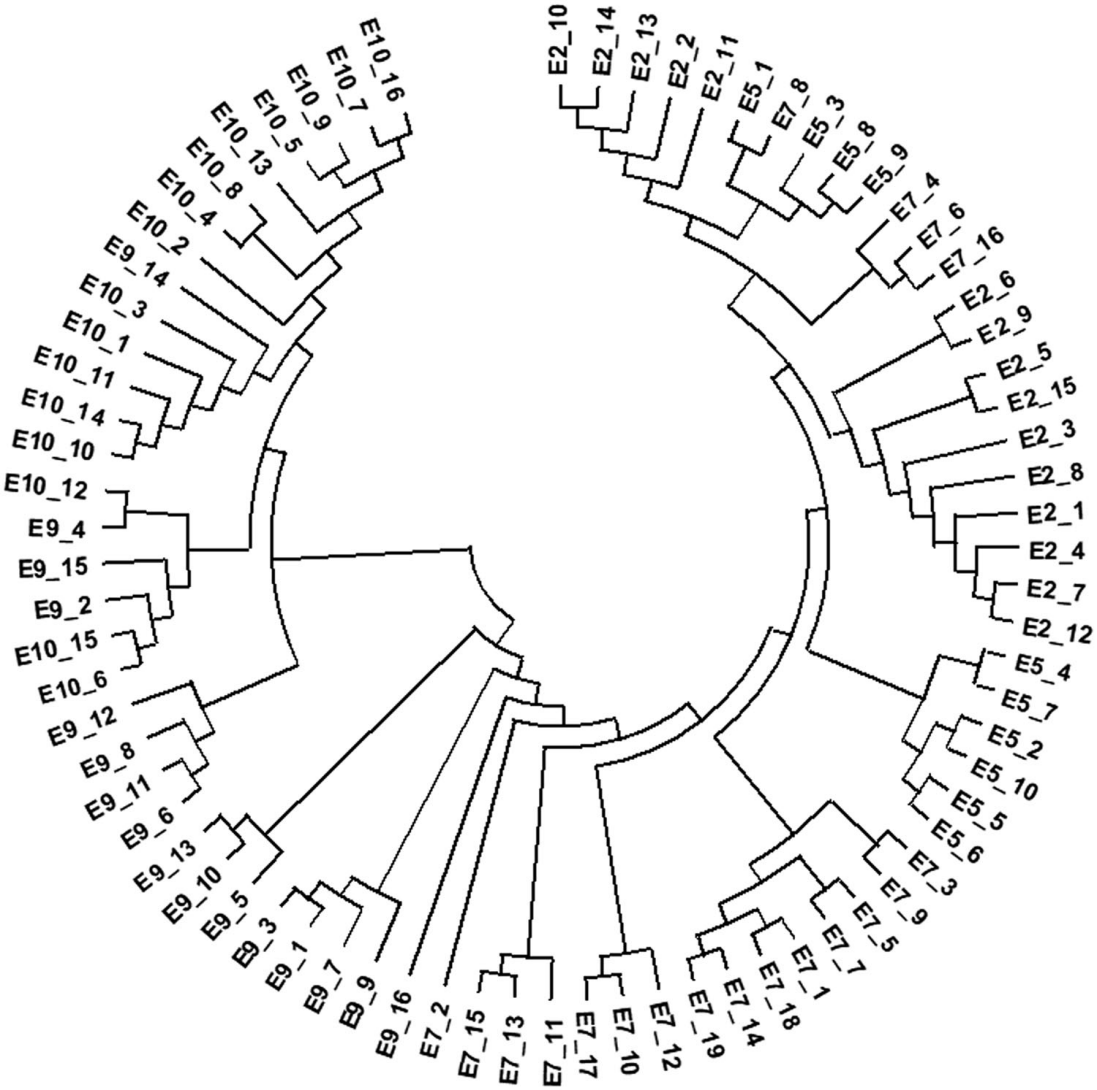
Phylogenetic tree of gp160 sequences derived from subject E. The data are adapted from a previous publication [8].

**Figure 6.**
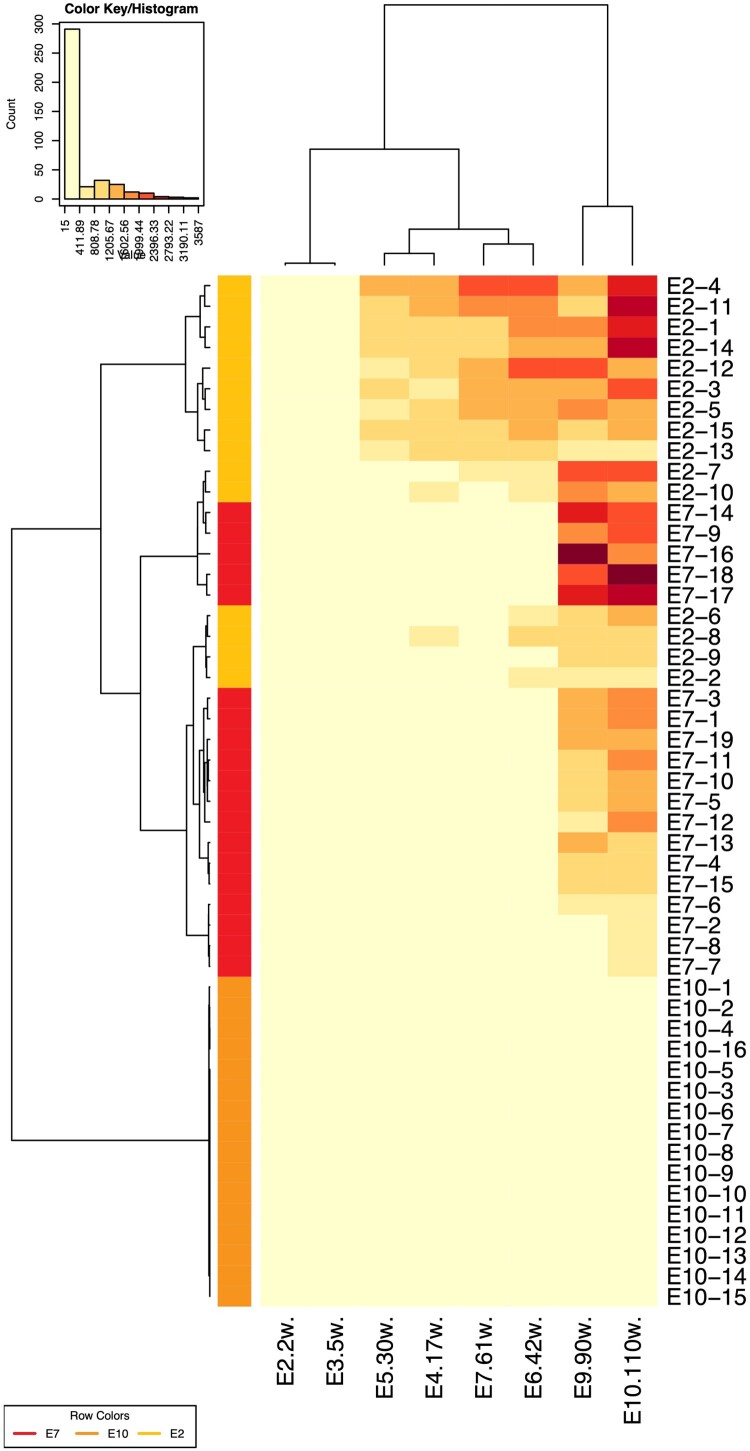
Heatmap to analyze the neutralization sensitivity between the plasma pools and the pseudoviruses at different time points. The data are adapted from a previous publication [8].

To study the relationship between viral evolution and the production of neutralizing antibody, the neutralizing antibodies from 75 subjects were tested against a set of four heterologous HIV-1 (three Tier 2 strains and SF162) pseudoviruses to select two subjects: one HIV-1-infected person who produced broad-spectrum neutralizing antibody (neutralizer, all four pseudoviruses could be neutralized) and one HIV-1-infected person who did not produce neutralizing antibody (non-neutralizer, none of the four could be neutralized) were selected from 75 HIV-1-infected individuals, as previously reported [8]. Both individuals were infected with subtype AE, with samples collected at different time points after infection to generate pseudoviruses. The samples from the neutralizer were collected 2, 5, 7, 9, and 10 months after infection, whereas viral samples from the non-neutralizer were collected 2, 6, 8, and 11 months following infection. Based on these samples, a total of 116 *env* genes were generated using the single genome amplification strategy (Figure 5) [13]. Compared with the non-neutralizer, *env* variants from the neutralizer demonstrated greater genetic evolution over time, such as a longer V1V2 region and more N-glycans, which is likely due to the presence of neutralizing antibodies facilitating viral evolution. Six neutralizing mAbs with well-defined epitope specificities (2F5, 4E10, 2G12, IgG1b12, PG9, and PG16) were also used to characterize the neutralization sensitivity of the pseudoviruses from the two different sources. Some strains derived from the neutralizer gradually became resistant to mAbs PG9/16 over the course of infection, whereas the virus strains derived from the non-neutralizer did not evolve towards greater neutralization resistance. To investigate the critical region or sites for neutralization-driven evolution, 18 chimeric pseudoviruses were constructed. This work revealed that some key sites in the C1-C3 region of gp160 were responsible for virus escape (Figure 6) [8].

## A systematic study of the role of N-glycosylation sites on the HIV-1 envelope protein on infectivity and antibody-mediated neutralization (100 pseudovirus strains)

Glycans on the HIV-1 Env protein play an important role in viral infection and the evasion of neutralization by antibodies. Despite the vast amount of literature on the N-linked glycosylation of gp120 and gp41, the impact of individual N-linked glycans on HIV-1 infectivity and antibody-mediated neutralization has not been systemically evaluated. To study the effect of glycosylation on viral infection and neutralization, 25 point mutations were introduced into the N-glycosylation sites, individually or in combination, of the envelope protein of a CRF07_BC strain FE (derived from the same sample as GX93.8 in the panel) to generate 64 mutants [6]. Then 18 patterns of mutations were introduced into another CRF07_BC strain SC19_15 and a subtype B strain YU-2 to confirm the observations [6,14]. Significantly altered infectivity was detected in some glycosylation mutants. Of the 100 mutant pseudoviruses, 24 mutations completely abolished infectivity and 33 mutations resulted in significantly diminished infectivity. Specifically, the infectivity of glycosylation mutants in the BC subtype appeared to be more significantly reduced than in subtype B [6]; mutations at N197 (residue positions on HIV-1 are based on HXB2 numbering throughout the manuscript) generally reduced viral infectivity, whereas N442Q mutations rendered the virus more infectious [14].

The remaining 43 mutant pseudoviruses were evaluated using neutralizing mAbs [6]. The results indicated that deletion of N197 (C2), N301 (V3), N442 (C4), and N625 (gp41) rendered pseudoviruses significantly more sensitive to antibodies targeting the anti-CD4 binding site and to mAbs targeting gp41. Furthermore, deletion of the N-glycan sites on the V4/V5 loop, the C2/C3/C4 region, and gp41 reduced the neutralizing sensitivity to mAb PG16, whereas deletion of N289 (C2) increased the neutralizing sensitivity to PG9 and PG16. It is also of note that deletion of partial N-glycan sites on V1, C3, and V5 increased neutralizing sensitivity to mAb gp41. The combined mutation of N197 and N463 is critical for virus neutralization sensitivity. The pseudovirus containing these two site mutations was 1000-fold more sensitive than the wild-type strain to the anti-CD4 binding site mAb VRC01/03 [14]. Although b12 belongs to the group of CD4bs mAbs, along with VRC01/03, these mAbs do not possess identical mechanisms to neutralize HIV-1 [15–17].

## Investigation of the key amino acid residues in HIV-1 Env protein regulating viral neutralization susceptibility to IgG1b12 (28 pseudovirus strains)

IgG1b12 (b12) was the first mAb with broad neutralizing activity against HIV-1 viruses to be studied in a non-human primate model [18]. Although the crystal structure of b12 mAb binding to the core of gp120 has been illustrated and the epitope on gp120 recognized by the b12 mAb has been defined [19], some key residues influencing b12 neutralization susceptibility, especially those not existing on the contact surface, have not been clearly determined, despite being critical for immunogen design. We found that a CRF07_BC pseudovirus strain from one infected patient was particularly sensitive to b12 mAb, with an IC_50_ value of 0.014 μg/ml (IC_50_ of SF162 is about 0.1 μg/ml). Different strains isolated from this individual showed different neutralization susceptibilities to b12 mAb. To avoid Taq polymerase-mediated template switching and errors, the single genome amplification method and high-fidelity polymerase were employed to amplify full-length *env* sequences. Based on these sequences, a quasispecies of 24 Env-pseudotyped viruses was constructed to examine the key residues affecting b12 neutralization susceptibility. A total of 11 site mutants were found to have IC_50_ values ranging from 0.014 μg/ml to more than 25 μg/ml against b12. Three Env mutants (182, 276, and 346) were found to vary significantly in their reactivities to b12 mAb. However, simultaneous mutation at these three sites did not alter the reactivity to b12. Moreover, three pseudoviruses were constructed with a V182L mutation in one strain of the same subtype and two strains of different subtypes (CRF08_BC and CRF01_AE). It has been reported that mutations at this site can alter the sensitivity of the same subtype and strain CRF08_BC with no effects on CRF01_AE [20]. Identifying vulnerable sites on HIV Env may provide the starting point for a structure-based approach to vaccine design. These sites of vulnerability are often targeted by neutralizing antibodies. Thus, identification of neutralizing antibodies and the elucidation of the epitopes and key residues targeted by these antibodies are powerful tools for immunogen design.

## Phenotypic analysis of drug-resistant isolates from China (46 pseudovirus strains)

Antivirals against HIV-1 are routinely tested in a BSL-3 laboratory setting since replication-competent virus is used in the assay. We set out to develop a pseudovirus-based approach that can be used for the analysis of drug resistance in a BSL-2 laboratory environment.

To construct a resistant HIV pseudovirus based on the pSG3Δ*env*-TZM-bl cell system, the HIV-1 backbone plasmid “pSG3Δ*env*” was modified by inserting the restriction endonuclease sites *Apa*I and *Age*I at sites 1553 and 3035, respectively, which corresponds to the HIV-1 protease (PR) and reverse transcriptase (RT) genes. A total of 16 drug-resistant HIV pseudoviruses were generated by replacing the PR and RT regions of pSG3Δ*env* with those derived from drug-resistant subjects [21]. The phenotypic drug-resistances of the 16 pseudoviruses covered three major anti-retroviral drugs, including: six nucleoside reverse transcriptase inhibitors (AZT, d4T, 3TC, ABC, TDF, and FTC), two non-nucleoside reverse transcriptase inhibitors (NVP and EFV), and four protease inhibitors (SQV, IDV, LPV, and NFV) [21]. This method could directly and precisely interpret the results when complex mutations were presented, and could also be used to screen new antiretroviral candidates.

The pSG3Δ*env* proviral clone contains a defective *vpu* gene and the plasmid tends to delete viral sequences during propagation in *Escherichia coli* [1,22]. To further simplify the cloning of the drug resistance gene, an alternative HIV-1 backbone plasmid pNL4-3.Luc.R-E was selected and modified by deleting a 788 bp fragment of the luciferase gene and replacing the PR and rRT genes (1479 bp) with the LacZ gene (447 bp) at endonucleases sites *Apa*I and *Age*I, respectively, which was designated as pNL4-3.Lac. The cloning efficiency with the LacZ replacement was largely enhanced. Six strains of pseudovirus were generated using this system, with one remaining drug-sensitive and the other five being nucleoside analogues and non-nucleoside reverse transcriptase inhibitors [23]. The comparability between pNL4-3.Lac and pSG3Δ*env* was confirmed by a head-to-head comparison using six pairs of strains with identical RT and PR regions [23]. Additional work was conducted to compare the two pseudoviral construction systems of NL4-3 and SG3 [21,24] with the traditional live virus assay, which employed live wild-type HIV-1 IIIB and MT4 cells. The results of the three methods were in good agreement, but pNL4-3.Lac was much easier to manipulate experimentally.

In addition to the evaluation of reverse transcriptase inhibitors and protease inhibitors by pseudoviruses, we established a method to evaluate HIV-1 membrane fusion inhibitors. Enfuvirtide (T20) is the leading compound of a new class of fusion inhibitor antiretroviral drugs. Most previous studies have only focused on investigating mutations based on sequence analysis of the HR1 region [25]. It has been reported that HR1 mutations located between amino acids 36 and 45 of the gp41 ectodomain are associated with T20 resistance (especially amino acids 35, 39, and 42) [25]. With the development of the pseudovirus inhibition assay, the biological function of the variants, especially susceptibility to T20, could be evaluated. Using the pseudovirus assay, the L33M mutation in gp41 HR1 was found to significantly increase the resistance of pseudovirus to T20 in a study including 27 pseudovirus strains derived from China [26]. Mutations in the HR1 region outside of the 10 amino acid motifs between residues 36 and 45 may therefore play a role in resistance to T20. Although T20 has not yet been used in China, the drug-resistance levels in naturally-infected individuals should be considered and amino acid mutations outside of the 36–45 residue region should not be ignored.

## Establishment of a pseudovirus-based neutralization assay using A3R5 cells (30 pseudovirus strains)

Recently, a new standardized and validated neutralization assay using the T-lymphoblastoid cell line A3R5 with physiological levels of CCR5 was established, which employed infectious molecular clones [27,28]. However, when A3R5 cells were used as the target cells for pseudovirus infection, the luciferase signal-to-noise ratio was too weak to yield robust results. By introducing the complete CMV promotor into the pSG3Δ*env* and replacing the promotor for Env protein in the expression plasmid, we generated a highly-efficient pseudovirus production system (pSG3Δ*env*.fluc + pCAG.Env), with which the titre of pseudoviruses could increase 10- to 100-fold [29]. To establish a HIV-1 pseudovirus neutralization assay based on A3R5 cells, 30 pseudoviruses were constructed using the highly-efficient pseudovirus production system [29,30]. Compared with the TZM-bl assay, the A3R5 assay based on pseudovirus showed significantly higher sensitivity, especially for the Tier 2 viruses, against which the A3R5 pseudovirus assay showed higher sensitivity for both neutralizing mAb (10-fold) and plasma (9-fold) samples [30]. The highly sensitive pseudovirus assay using more physiological target cells could serve as an alternative to the TZM-bl assay for the evaluation of vaccine-induced neutralizing antibodies and the identification of the correlates of protection.

## Conclusion

Our laboratory constructed 462 pseudoviruses with viral genes isolated in China; these pseudoviruses covered the majority of the epidemic subtypes and recombinant viruses. All pseudoviruses are being widely shared among interested scientists conducting research on HIV-1 and those developing vaccines and antiviral drugs against HIV-1, thereby improving consistency, comparability, and reproducibility across laboratories. Clearly, continued efforts should be made to generate and improve the pseudovirus repository, given the evolving nature of HIV-1 viruses.

## Supplementary Material

Supplemental Material
