# Supplementary material for: HIV-1 pseudoviruses constructed in China regulatory laboratory
Source: Emerg Microbes Infect. 2019 Dec 20;9(1):32–41. doi: 10.1080/22221751.2019.1702479 (PMC6968579; doi:10.1080/22221751.2019.1702479)
Supplement: Supplemental Material [file TEMI_A_1702479_SM0040.doc]

Supplementary table 1. Basic information and characterization of 124 HIV-1 clones

| number | Clone | Location | Subtype | Accession number in NCBI |
| --- | --- | --- | --- | --- |
| 1 | HN6A.40 | Henan | B | MN642095 |
| 2 | HN18A.41 | Henan | B | MN642097 |
| 3 | HN15A.27 | Henan | B | MN642096 |
| 4 | HN40.40 | Henan | B | MN642098 |
| 5 | BJYA105.5 | Beijing | B | MN617383 |
| 6 | BJYA109.9 | Beijing | B | MN617384 |
| 7 | BJYA119.6 | Beijing | B | MN617385 |
| 8 | BJYA144.8 | Beijing | B | MN617386 |
| 9 | BJYA174.16 | Beijing | B | MN617387 |
| 10 | BJYA180.8 | Beijing | B | MN617388 |
| 11 | BJYA187.8 | Beijing | B | MN617389 |
| 12 | BJYA192.4 | Beijing | B | MN617390 |
| 13 | BJYA194.5 | Beijing | B | MN617391 |
| 14 | B01 | Hebei | B' | EU363825 |
| 15 | B02 | Gansu | B' | EU363826 |
| 16 | B03 | Hebei | B' | EU363827 |
| 17 | B04 | Hebei | B' | EU363828 |
| 18 | B05 | Hubei | B' | EU363829 |
| 19 | B06 | Beijing | B' | EU363830 |
| 20 | HeB5.10 | Hebei | B' | MN617374 |
| 21 | HuB32.3 | Hubei | B' | MN617375 |
| 22 | BJ33.11 | Beijing | B' | MN642094 |
| 23 | BJ39.14 | Beijing | B' | MN617376 |
| 24 | HeB87.2 | Henan | B' | MN617377 |
| 25 | BJ168.17 | Beijing | B' | MN617378 |
| 26 | BJ171.13 | Beijing | B' | MN617379 |
| 27 | BJ175.4 | Beijing | B' | MN617380 |
| 28 | BJ176.1 | Beijing | B' | MN617381 |
| 29 | HeB46.6 | Hebei | B' | MN617382 |
| 1 | AE01 | Gangdong | CRF01_AE | EU363849 |
| 2 | AE02 | Yunnan | CRF01_AE | EU363850 |
| 3 | AE03 | Shanghai | CRF01_AE | EU363851 |
| 4 | BJ17A.6 | Beijing | CRF01_AE | GU475028 |
| 5 | BJ3.4 | Beijing | CRF01_AE | GU475040 |
| 6 | BJ5.11 | Beijing | CRF01_AE | GU475024 |
| 7 | BJ6.17 | Beijing | CRF01_AE | GU475029 |
| 8 | BJX4.6 | Beijing | CRF01_AE | GU475020 |
| 9 | GX11.13 | Guangxi | CRF01_AE | GU475042 |
| 10 | GX13.7 | Guangxi | CRF01_AE | GU475037 |
| 11 | GX142.2 | Guangxi | CRF01_AE | GU475031 |
| 12 | GX155.55 | Guangxi | CRF01_AE | GU475043 |
| 13 | GX2010.36 | Guangxi | CRF01_AE | GU475026 |
| 14 | GX24.8 | Guangxi | CRF01_AE | GU475015 |
| 15 | GX25.29 | Guangxi | CRF01_AE | GU475030 |
| 16 | GX28.31 | Guangxi | CRF01_AE | GU475017 |
| 17 | GX34.21 | Guangxi | CRF01_AE | GU475022 |
| 18 | GX35.33 | Guangxi | CRF01_AE | GU475023 |
| 19 | GX54.6 | Guangxi | CRF01_AE | GU475045 |
| 20 | GX68.5 | Guangxi | CRF01_AE | GU475044 |
| 21 | GX71.18 | Guangxi | CRF01_AE | GU475016 |
| 22 | GX72.27 | Guangxi | CRF01_AE | GU475035 |
| 23 | GX73.29 | Guangxi | CRF01_AE | GU475038 |
| 24 | GX74.20 | Guangxi | CRF01_AE | GU475021 |
| 25 | GX81.43 | Guangxi | CRF01_AE | GU475014 |
| 26 | GX83.47 | Guangxi | CRF01_AE | GU475027 |
| 27 | GX88.47 | Guangxi | CRF01_AE | GU475018 |
| 28 | GX8C.31 | Guangxi | CRF01_AE | GU475039 |
| 29 | GX90.1 | Guangxi | CRF01_AE | GU475019 |
| 30 | GX91.2 | Guangxi | CRF01_AE | GU475013 |
| 31 | GZ187.10 | Guangzhou | CRF01_AE | GU475036 |
| 32 | HuB199.1 | Hubei | CRF01_AE | GU475025 |
| 33 | SH188.6 | Shanghai | CRF01_AE | GU475041 |
| 34 | SH6.81 | Shanghai | CRF01_AE | GU475032 |
| 35 | SHX335.24 | Shanghai | CRF01_AE | GU475033 |
| 36 | SHX346.60 | Shanghai | CRF01_AE | GU475034 |
| 37 | YN192.31 | Yunan | CRF01_AE | GU475046 |
| 38 | BJYA2.1.1 | Beijing | CRF01_AE | MN617372 |
| 39 | BJYA10.1.3 | Beijing | CRF01_AE | MN617373 |
| 1 | BC02 | Xinjiang | CRF07_BC | EU363832 |
| 2 | BC03 | Xinjiang | CRF07_BC | EU363833 |
| 3 | BC04 | Sichuan | CRF07_BC | EU363834 |
| 4 | BC05 | Sichuan | CRF07_BC | EU363835 |
| 5 | BC07 | Yunnan | CRF07_BC | EU363837 |
| 6 | BC09 | Beijing | CRF07_BC | EU363839 |
| 7 | BC10 | Yunnan | CRF07_BC | EU363840 |
| 8 | BC11 | Yunnan | CRF07_BC | EU363841 |
| 9 | BC12 | Sichuan | CRF07_BC | EU363842 |
| 10 | BC14 | Beijing | CRF07_BC | EU363844 |
| 11 | BC15 | Beijing | CRF07_BC | EU363845 |
| 12 | BC16 | Beijing | CRF07_BC | EU363846 |
| 13 | BC18 | Xinjiang | CRF07_BC | EU363848 |
| 14 | HB5-3 | Hebei | CRF07_BC | HQ326124 |
| 15 | BJ22-5 | Beijing | CRF07_BC | HQ326125 |
| 16 | BJ24-3 | Beijing | CRF07_BC | HQ326126 |
| 17 | SC11-59 | Sichuan | CRF07_BC | HQ326127 |
| 18 | SC17-32 | Sichuan | CRF07_BC | HQ326128 |
| 19 | SC19-15 | Sichuan | CRF07_BC | HQ326129 |
| 20 | SC20-15 | Sichuan | CRF07_BC | HQ326130 |
| 21 | SC21-28 | Sichuan | CRF07_BC | HQ326131 |
| 22 | SC22-16 | Sichuan | CRF07_BC | HQ326132 |
| 23 | SC24-40 | Sichuan | CRF07_BC | HM991498 |
| 24 | XJ16-6 | Xinjiang | CRF07_BC | HQ326133 |
| 25 | XJ47-5 | Xinjiang | CRF07_BC | HQ326134 |
| 26 | XJ50-6 | Xinjiang | CRF07_BC | HQ326135 |
| 27 | XJ180-29 | Xinjiang | CRF07_BC | HQ326136 |
| 28 | YN99r-5 | Yunan | CRF07_BC | HQ326137 |
| 29 | YN108r-4 | Yunan | CRF07_BC | HQ326138 |
| 30 | YN148r-9 | Yunan | CRF07_BC | HQ326139 |
| 31 | GX33m-25 | Guangxi | CRF07_BC | HQ326141 |
| 32 | GX45-57 | Guangxi | CRF07_BC | HQ326143 |
| 33 | GX79-7 | Guangxi | CRF07_BC | HQ326145 |
| 34 | GX84-59 | Guangxi | CRF07_BC | HQ326146 |
| 35 | BJ23-1 | Beijing | CRF07_BC | MN617361 |
| 36 | YN189-52 | Yunan | CRF07_BC | MN617363 |
| 37 | GX93-8 | Guangxi | CRF07_BC | MN617364 |
| 38 | YN112R.8 | Yunan | CRF07_BC | MN617365 |
| 39 | XJ179.47 | Xinjiang | CRF07_BC | MN617368 |
| 40 | XJ180.16 | Xinjiang | CRF07_BC | MN617369 |
| 41 | SC14.49 | Sichuan | CRF07_BC | MN617370 |
| 42 | SC18.26 | Sichuan | CRF07_BC | MN617371 |
| 43 | GZ30.6 | Guangzhou | CRF07_BC | MN617392 |
| 44 | GZ36.5 | Guangzhou | CRF07_BC | MN617393 |
| 45 | GZ38.2 | Guangzhou | CRF07_BC | MN617394 |
| 46 | BC01 | Yunnan | CRF08_BC | EU363831 |
| 47 | BC06 | Yunnan | CRF08_BC | EU363836 |
| 48 | BC08 | Yunnan | CRF08_BC | EU363838 |
| 49 | BC13 | Yunnan | CRF08_BC | EU363843 |
| 50 | BC17 | Yunnan | CRF08_BC | EU363847 |
| 51 | YN177-1 | Yunan | CRF08_BC | HQ326140 |
| 52 | GX43-2 | Guangxi | CRF08_BC | HQ326142 |
| 53 | GX75-20 | Guangxi | CRF08_BC | HQ326144 |
| 54 | BJ28-13 | Beijing | CRF08_BC | MN617362 |
| 55 | SC12.10 | Sichuan | CRF08_BC | MN617366 |
| 56 | YN6.23 | Yunan | CRF08_BC | MN617367 |
